# Genome-wide identification of RNA recognition motif (RRM1) in *Brassica rapa* and functional analysis of RNA-binding protein (*BrRBP)* under low-temperature stress

**DOI:** 10.1186/s12870-023-04639-4

**Published:** 2023-12-07

**Authors:** Li Ma, Xiaolei Tao, Wangtian Wang, Jintang Jiao, Yuanyuan Pu, Gang Yang, Lijun Liu, Yan Fang, Junyan Wu, Wancang Sun

**Affiliations:** 1 State Key Laboratory of Aridland Crop Science, Gansu Agricultural University, Lanzhou, 730070 China; 2 College of Agronomy, Gansu Agricultural University, Lanzhou, 730070 China

**Keywords:** Winter *Brassica rapa*, RRM1 gene family, Gene expression, Low-temperature stress, Gene function

## Abstract

**Background:**

The RNA recognition motif (RRM) is primarily engaged in the processing of mRNA and rRNA following gene transcription as well as the regulation of RNA transport; it is critical in preserving RNA stability.

**Results:**

In this study, we identified 102 members of the RRM1 gene family in *Brassica rapa*, which were dispersed across 10 chromosomes with the ninth chromosome being the most extensively distributed. The RRM1 gene family members of *Brassica rapa* and *Arabidopsis thaliana* were grouped into 14 subclades (I–XIV) using phylogenetic analysis. Moreover, the results of transcriptome analysis and RT-qPCR indicated that the expression of Brapa05T000840 was upregulated in the cultivars ‘Longyou 7’ and ‘Longyou 99’ following exposure to cold stress at a temperature of 4 °C for 24 h. The levels of expression in the leaves and growth cones of the ‘Longyou 7’ variety were found to be significantly higher than those observed in the ‘Longyou 99’ variety under conditions of low temperature and NaCl stress. It illustrates the involvement of the RRM1 gene in the physiological response to both low temperature and salt stress. In addition, it was observed that the survival rate of transgenic *BrRBP* (Brapa05T000840) *Arabidopsis thaliana* plants was notably higher compared to that of wild-type plants when subjected to varying durations of low temperature treatment. Furthermore, the expression of the *BrRBP* gene in transgenic plants exhibited an upward trend as the duration of low temperature treatment increased, reaching its peak at 24 h. The in-vivo enzymatic activity of reactive oxygen species-scavenging enzymes were found to be significantly elevated in comparison to wild-type plants, suggesting that the *BrRBP* gene may enhance the cold tolerance of *Arabidopsis thaliana*.

**Conclusions:**

This study offers a significant foundation for comprehending the regulation mechanism of the RRM1 gene family in winter *Brassica rapa* subjected to cold stress, as well as for finding key genes associated with cold resistance.

**Supplementary Information:**

The online version contains supplementary material available at 10.1186/s12870-023-04639-4.

## Background

The northern regions of China are mostly located at mid-latitudes where light energy is abundant and the temperature difference between day and night is large. Summers are short and hot, winters are long and very cold, and the climate is characterized by drought and cold. In recent years, global climatic conditions have become increasingly dramatic, with extreme weather events such as frost and cold damage occurring frequently, causing varying degrees of damage to the growth and yield of many crops [1]. The winter rapeseed (*Brassica rapa* L.) has excellent cold resistance and adaptability, with superior yields among similar oil crops, and has developed into a major cultivation type in the northern regions of China. The cold resistance of winter rapeseed has become an important agronomic indicator, which can judge the degree of winter rapeseed variety excellence and is also a prerequisite for stable and high yields of winter rapeseed in the dry and cold regions of northwest China [2, 3]. Therefore, the analysis of the cold resistance mechanism of winter rapeseed can provide an effective basis for germplasm selection and variety breeding.

In eukaryotic cells, gene expression is tightly regulated at both the transcriptional and post-transcriptional levels of translation. Transcriptional regulation was once considered the main regulatory mechanism of gene expression. Various proteins are involved in the post-transcriptional regulation of genes [4]. The most important and widely studied ones are RNA-binding proteins with the structural domain of the RNA recognition motif (RRM) [5, 6], RRM plays a role in RNA metabolism, including Pre-RNA splicing, RNA stability, polyadenylation, transcriptional and post-transcriptional gene silencing, and RNA editing, It is the most abundant protein domain [7–9].

The RRM, also known as the RNA-binding domain (RND) or ribonucleoprotein domain (RNP), is a region of approximately 80 to 90 amino acids containing many very conserved residues, some of which cluster into two short sub-motifs for RNA-binding ribonucleoproteins, RNP-1 (octamer) and RNP-2 (hexamer) [7, 10, 11]. RNP-1 and RNP-2 sequences contain aromatic and basic residues associated with nucleic acid interactions. Nonconserved residue sequences provide additional contacts for specific RNA recognition by deletion and site-directed mutagenesis using the spliceosomal protein U1 small nuclear ribonucleoprotein 70 K (U1snRNP-70 K) ~2. Multiple RRM family members in proteins can bind multiple RNA molecules simultaneously, and all RRM proteins share a common fold and a similar protein-RNA interface [9, 12]. The RRM structural domain is not only in numerous nuclear inhomogeneous ribonucleoproteins (hnRNPs) but also in proteins that regulate selective splicing; it is a component of small ribonucleoproteins. In eukaryotic proteins, RRMs are commonly found in multiple copies of the protein (44%, two to six RRMs) or bound to other accessory domains (21%); the most abundant binding are the zinc finger domain of the CCCH and CCHC types (21%), the C-terminal domain of polyadenylate binding protein (PABP or PABC, 10%), and the WW domain (9%) [11, 13, 14].

Studies have shown that RRM is involved in protein transport to the nucleus [15, 16]. By associating with different types of protein domains, RRMs can regulate their RNA-binding affinity and specificity, diversifying their biological functions. Hong et al. showed that the volume of various tissues and organs, such as leaves, glumes, pollen, and stigma of positive transgenic plants overexpressing the RRM1 domain and RRM2 domain increased compared with the corresponding tissues and organs of control plants [17, 18]. The RRM domain differentially affects the whole function of the *Arabidopsis thaliana* AtSF1 protein under different experimental conditions. The deletion of the RRM domain affects ATSF1-mediated flowering time control but not abscisic acid sensitivity during seed germination [19].

RNA binding proteins (RBPs) are widely present in organisms through the regulation of related genes at the post-transcriptional level. It recognizes and binds functional sequences of related regulatory genes, and affects the processing and modification of genes at the RNA level, which in turn regulates the transcriptional expression level of this gene [20]. In plant research, RBP affects plant growth and development by binding RNA. RBP controls mRNA localization, which in turn regulates the transcriptional expression levels of the corresponding genes in the organism, and performs the corresponding protein modifications and subcellular localization to improve the resistance of plants to environmental and other stresses [21]. RNA-binding protein (RBP) is involved in *Arabidopsis thaliana* growth metabolism, development, cold stress, drought stress, and other processes. Recent research has shown that RBM25, one of the RNA binding proteins in *Arabidopsis thaliana*, can play a role in stress response, and regulation of gene expression at the post-transcriptional level is mainly achieved by proteins containing well-defined sequences involved in RNA binding [20, 21]. With evolution, all eukaryotic gene lineages have retained the genes required to express the basic mechanisms of post-transcriptional regulation [22, 23]. Glycine-rich RNA-binding proteins are glycine-rich proteins (GRPs), which contain an N-terminal RRM and a C-terminal glycine-rich structural domain and an RRM and C-terminal glycine-rich domain at their N-terminus. They play a key role in adapting organisms to biotic and abiotic stresses, including those caused by pathogenesis, osmotic, saline, and oxidative environmental alterations, and temperature changes [24–26].

Most studies of the RRM1 domain have been focused on rice, *Mesembryanthemum crystallinum* L., maize, and *Arabidopsis thaliana* [5, 27–32], but few studies on the *RRM1* gene in *Brassica rapa* have been reported. Therefore, identifying and analyzing the RRM1 gene family in *Brassica rapa* can provide data reference and a theoretical basis for further studies on the biological functions of RRM1 gene family members in *Brassica rapa*. Based on the cold stress genome and transcriptome in winter *Brassica rapa*, we identified and analyzed the RRM1 gene family members in *Brassica rapa* using bioinformatics methods. We investigated their gene structure, physicochemical properties, phylogenetic relationships, gene duplication events, etc., analyzed their differential expression patterns under corresponding cold stress, and transformed the *BrRBP* gene into *Arabidopsis thaliana* to explore its functions. This study provides important information to further reveal the regulatory mechanism of the RRM1 gene family under cold stress in *Brassica rapa* and the identification of key cold-resistance genes. This study aimed to provide a theoretical basis for the subsequent study of the function of the *RRM1* gene in *Brassica rapa*.

## Results

### Chromosome mapping and protein physicochemical properties analysis of the RRM1 gene family in *Brassica rapa*

**Fig. 1:**
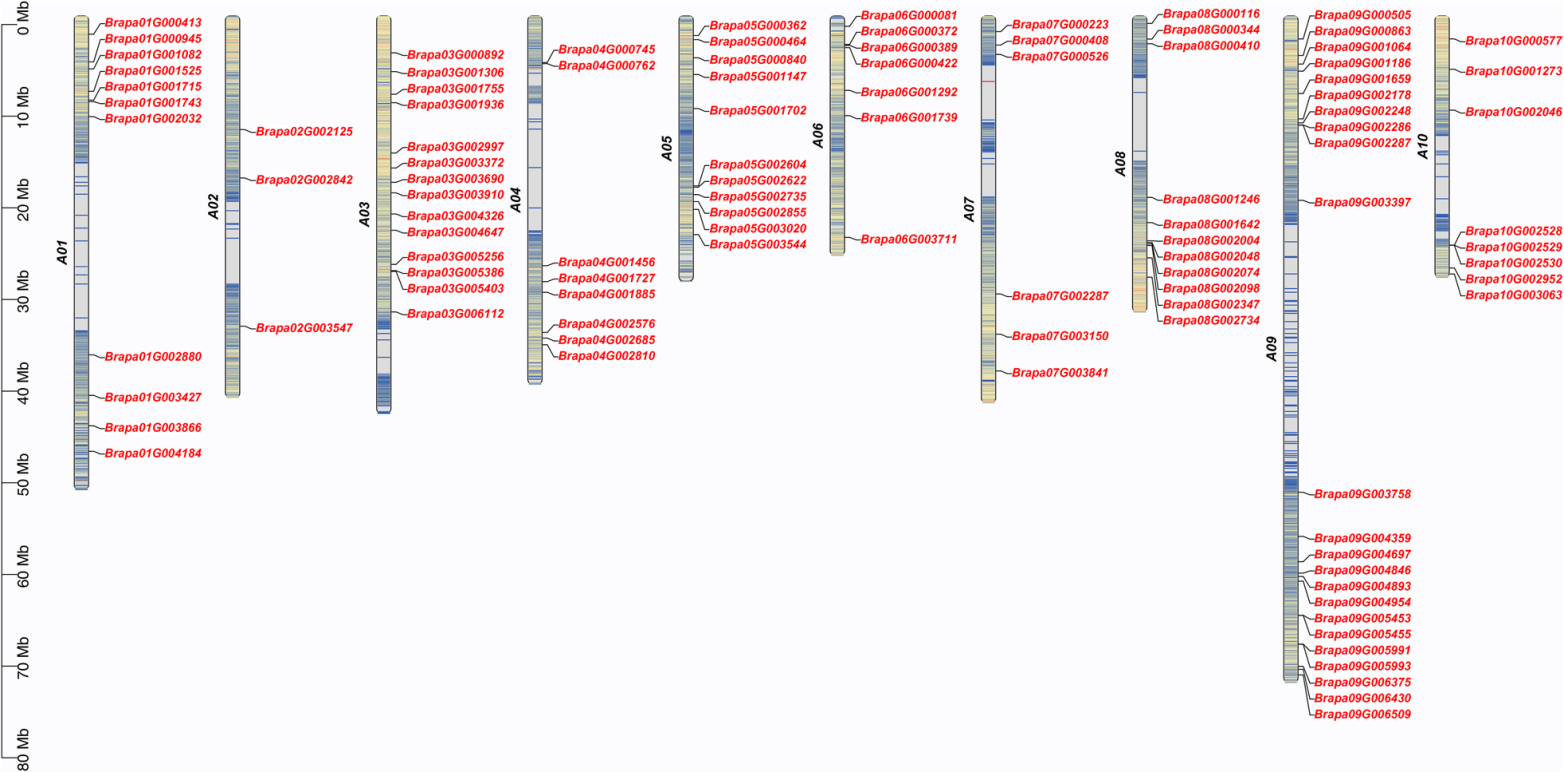
The chromosome location of the RRM1 gene family in *Brassica rapa*

In total, 102 RRM1 gene family members were obtained through a homology search. Chromosome localization analysis of the RRM1 gene family members (Fig. 1) revealed that 102 members of the family were distributed on 10 chromosomes of *Brassica rapa*, among which Chromosome A09 had the higher gene concentration (23). Chromosome A02 had the lower gene concentration (3), and the remaining gene members were unevenly located on the remaining chromosomes.

The predicted physicochemical properties of the protein showed that RRM1 encodes amino acids in the range of 251aa (Brapa09T002286, Brapa09T002287)–1779aa (Brapa01T001082). The relative molecular mass ranged from 27.32 kD (Brapa09T001186, Brapa09T001659) to 201.14 kD (Brapa01T001082), PI from 4.54 (Brapa09T006509) to 11.01 (Brapa09T006430), and exon number from 2 (Brapa03T001755, Brapa04T002576, Brapa05T001147, Brapa08T002347, and Brapa09T004893) to 26 (Brapa01T002032). Subcellular localization predictions showed that RRM1 gene family members were mainly localized in chloroplasts, mitochondria, cytoplasmic stroma, and the nucleus (Table S1).

### Phylogenetic analysis of the RRM1 gene family members in *Brassica rapa*

**Fig. 2:**
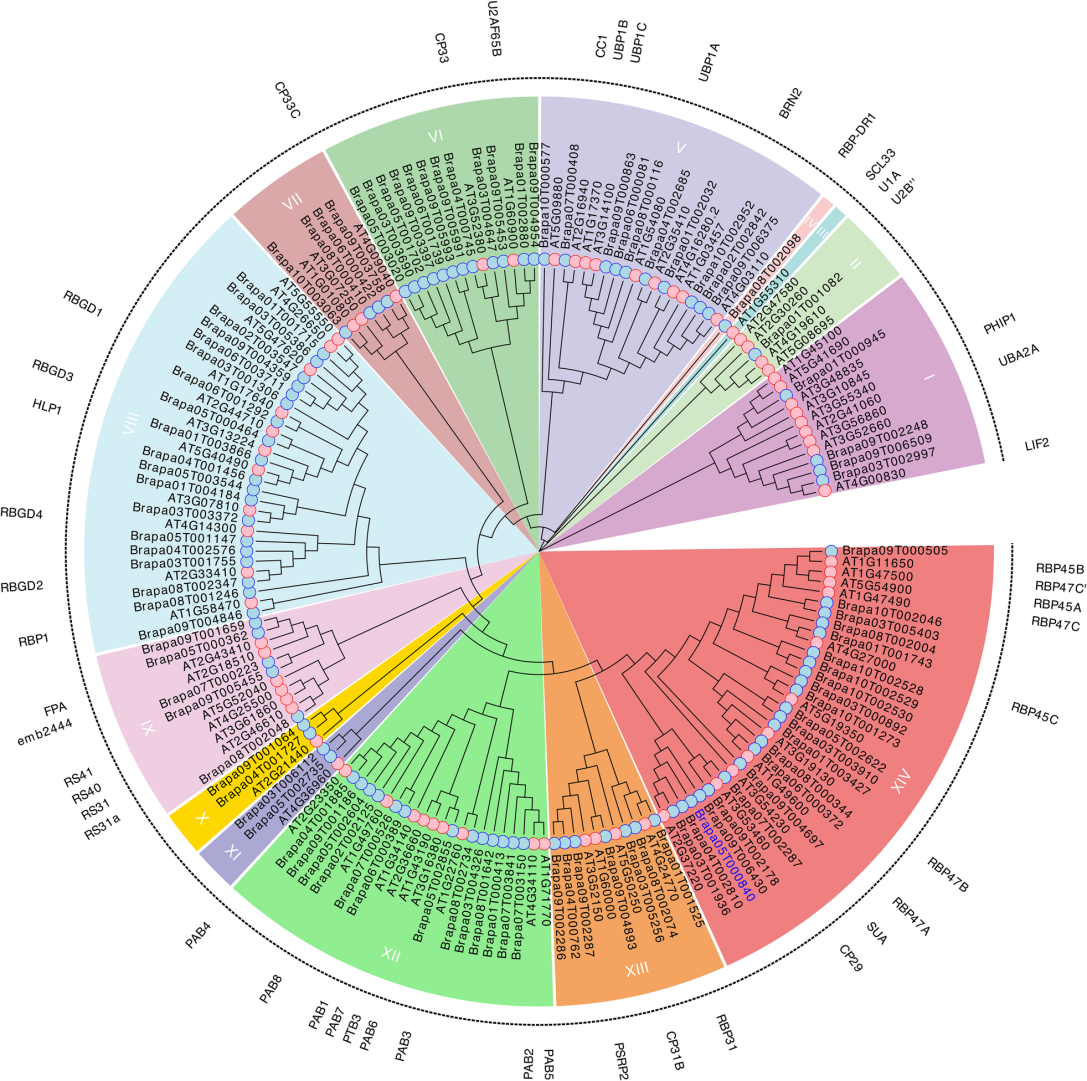
The phylogenetic tree of RRM1 in *Brassica rapa* and *Arabidopsis thaliana* was constructed using the maximum likelihood method. The 14 subfamilies are colored differently. blue circles represent *Brassica rapa*, The pink circles represent *Arabidopsis thaliana*. Letter abbreviations in the outer circle indicate known and named members of the RRM1 family in *Arabidopsis thaliana*

To further understand the evolutionary and functional characteristics of the RRM1 family members of *Brassica rapa*, we selected the RRM1 family members of *Arabidopsis thaliana* of the *cruciferae* family and constructed a common phylogenetic tree by the maximum likelihood (ML) method. And the names of functionally known *Arabidopsis thaliana* RRM1 family members were displayed in the outer circle. In addition, the *Brassica rapa* BrRBP (RNA binding protein CP29b, Brapa05T000840) was successfully distributed in the same subfamily as Arabidopsis CP29, indicating the reliability of the constructed evolutionary tree (Fig. 2). The phylogenetic relationship showed that the RRM1 family of *Brassica rapa* and *Arabidopsis thaliana* was divided into 14 subgroups (Ι–XIV). The number of genes distributed in subgroup XIV was the largest (33). The members of the RRM1 gene family in *Brassica rapa* were not distributed in subgroup III; they were unevenly dis-tributed in the 13 subgroups. The RRM1 gene family members of Arabidopsis thaliana were not distributed in subgroup IV but unevenly distributed in other subgroups. The RRM1 gene of *Brassica rapa* and *Arabidopsis thaliana* was relatively conservative in the evolution process, with certain similarities in function.

### Gene structure and motif sequence analysis of the RRM1 gene family in *Brassica rapa*

**Fig. 3:**
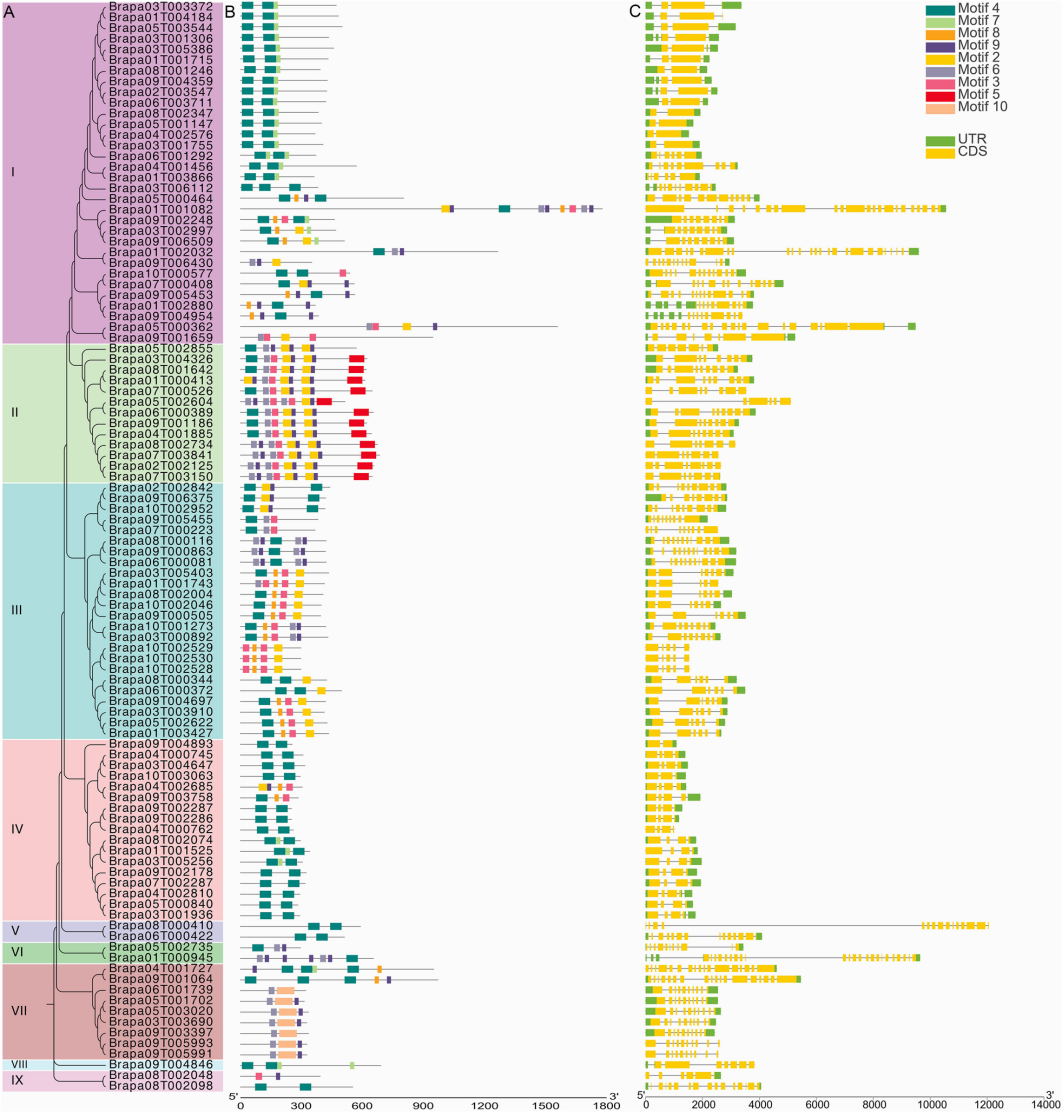
RRM1 gene family sequence analysis in *Brassica rapa*. **A** The 102 members are assigned to 9 subfamilies. **B** Motif analysis of the RRM1 gene family in *Brassica rapa*. Each colored box represents a motif, and the gray line represents a non-conserved sequence. **C** Gene structure of the RRM1 gene family in *Brassica rapa*. Exons and introns are represented by boxes and gray lines

The RRM1 gene family *of Brassica rapa* was individually clustered into 9 subfamilies (Fig. 3A). The conserved protein motifs of 102 members of the RRM1 family were analyzed using MEME software, and 9 conserved protein motifs (Motifs) were predicted (Fig. 3B). Most members of the same subfamily contained the same Motif composition. Motif 4 matched in subfamilies I–IX, this suggests that motif 4 may be a conserved and important motif in the RNA recognition motif. Motif 7 matched in subfamilies I, IV, VII, and VIII, Motif 8 matched in subfamilies I, III, IV, and VII, Motif 9 matched in I, II, III, IV, VI, VII, and IX, Motif 2 matched in I–IV, Motif 6 matched in I, II, III, VI, and VII, Motif 3 matched in I–IV and IX, Motif 5 matched only in II, and Motif 10 matched only in VII. In addition, subfamilies I contain the most motifs, including motifs 4, 9, 6, 3, and 2, implying that specific motifs may give RRM1 a specific function. Overall, sequences with similar motif structures clustered together, indicating the reliability of phylogenetic tree classification.

In addition, to understand the sequence features of RRM1, we analysed their gene structure, including the number of introns and exons. Generally, the number of exons was redundant to the number of introns (Fig. 3C). Among the 102 RRM1 members, a total of 87 members (85.29%) had more than 4 exons, 15 (14.71%) had 2 exons, and 68 (66.67%) had more than 6 exons. On the whole, some subfamilies (I and IV) have the same number of introns and exons, reflecting their structural and functional similarity, and most subfamilies have widely varying numbers of introns and exons, reflecting their structural and functional diversity.

### Collinearity analysis of the RRM1 gene family

**Fig. 4:**
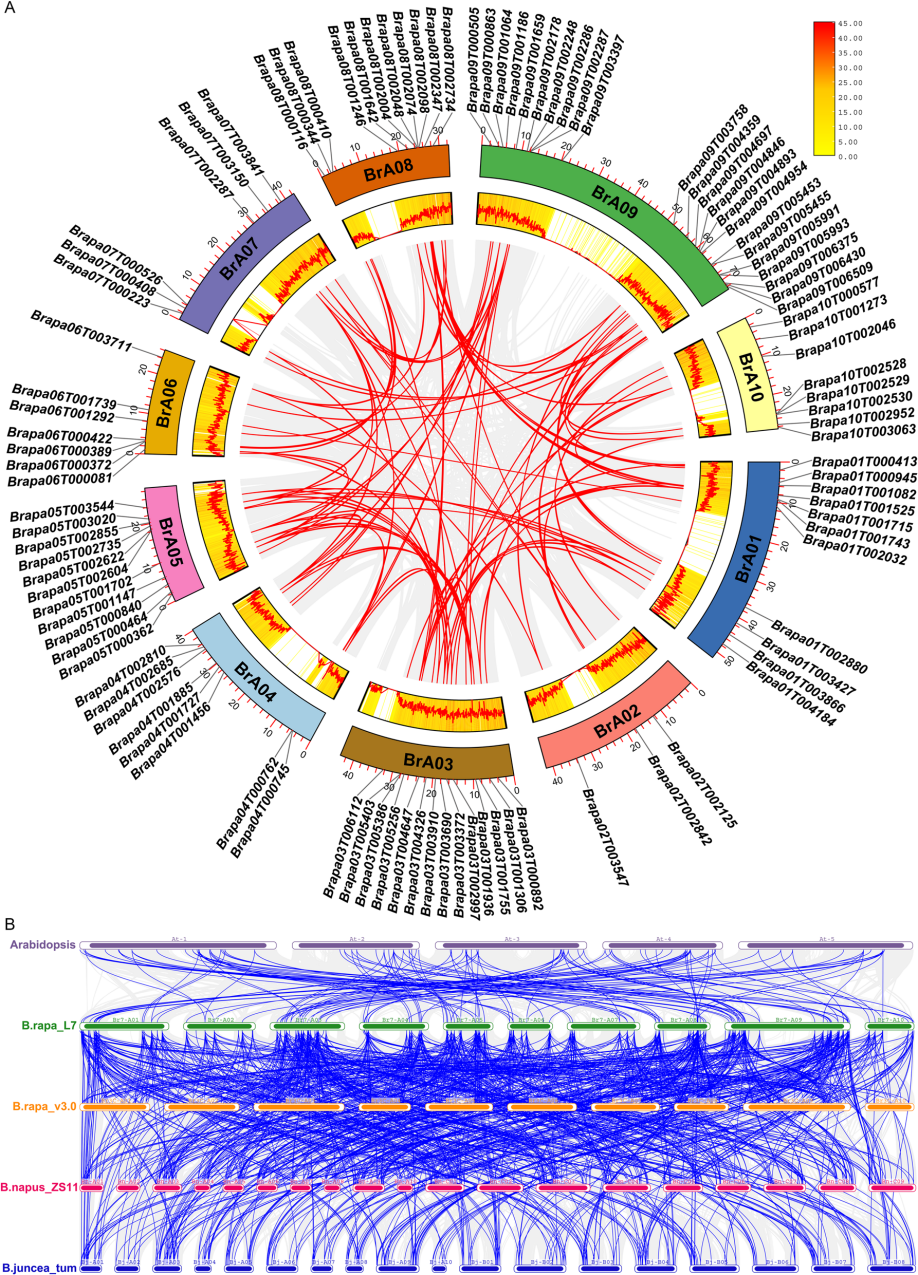
Collinearity analysis of the RRM1 gene family. **A** Collinearity of RRM1 gene family members in *Brassica rapa* is indicated by red lines. The different coloured modules on the outer layer represent the different chromosomes, and the middle layer represents the gene density line graph and heat map on the different chromosomes. **B** Collinearity of the RRM1 gene in *Brassica rapa*, *Arabidopsis thaliana*, *Brassica napus*, and *Brassica juncea* is indicated by the blue line. The different box colors represent the distribution of the chromosome where the gene is located

The gene replication events of the RRM1 family in *Brassica rapa* were studied to understand further the amplification mechanism of the RRM1 gene family. The intra-genomic collinear analysis in *Brassica rapa* showed 89 pairs of collinear genes belonging to segmental repetition, indicating that segmental repetition played a key role in the amplification of the RRM1 gene family in *Brassica rapa*. The genes with a collinear relationship were unevenly distributed on 10 chromosomes. There was a collinear relationship between one RRM1 gene and multiple RRM1 genes, such as Brapa02T002125 and Brapa07T003841, Brapa07T003150, Brapa08T002734 at the same time (Fig. 4A, Table S2).

Analysis of the colinear relationships of the RRM1 gene family genes in *Arabidopsis thaliana*, *Brassica rapa*, *Brassica napus*, and *Brassica juncea* revealed that the RRM1 gene in *Brassica rapa* was a colinear gene with the RRM1 genes in *Arabidopsis thaliana*, *Brassica napus*, and *Brassica juncea*, indicating that the gene was highly conserved during evolution (Fig. 4B, Table S3).

### Expression analysis of the RRM1 gene family under cold stress

**Fig. 5:**
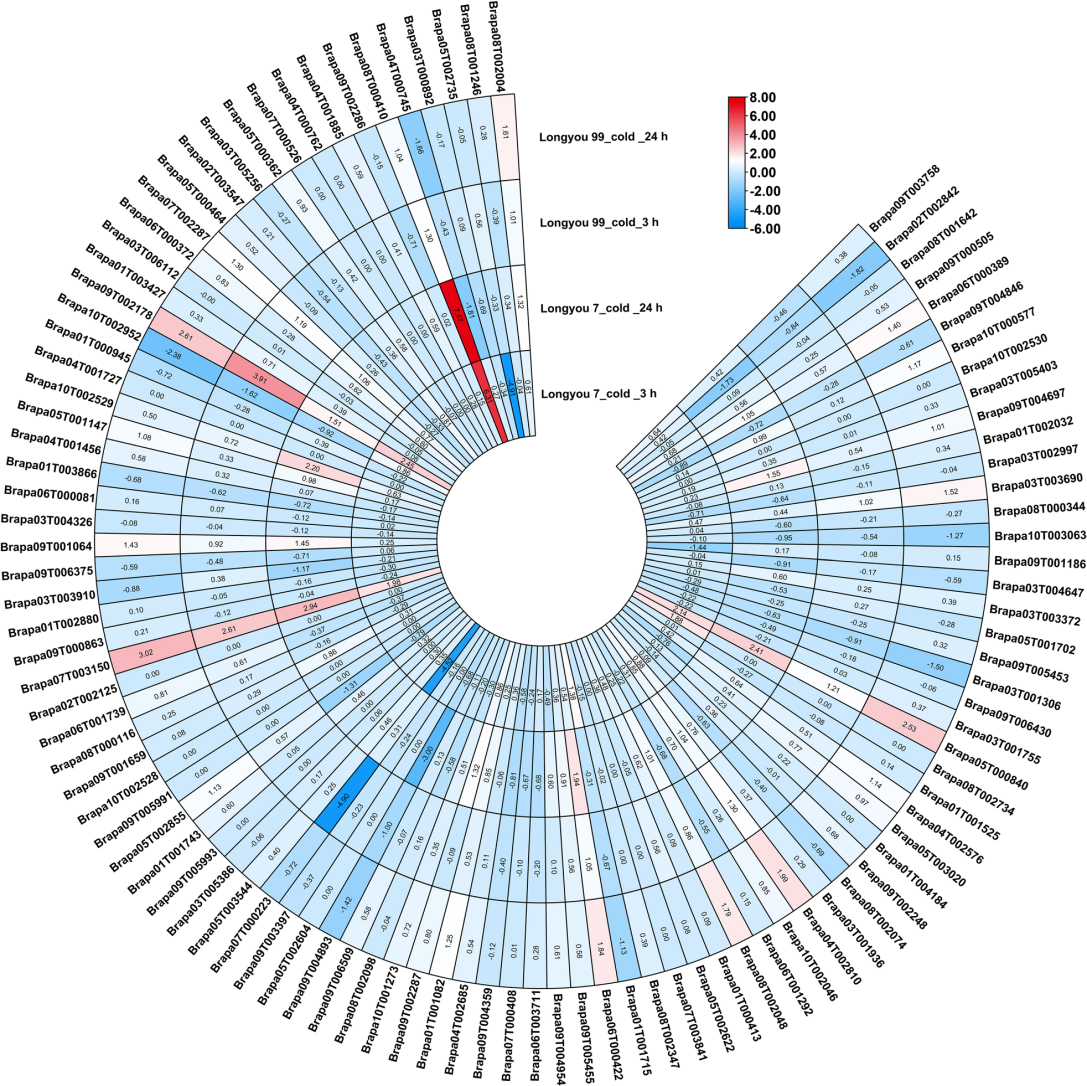
Expression pattern of the RRM1 gene family members in different winter *Brassica rapa* Varieties under cold stress. Heat maps were expressed using log2 values for each gene. The color scale represents the relative expression levels from low (blue) to high (red)

Based on the transcriptome data of two *Brassica rapa* varieties, ‘Longyou 7’ and ‘Longyou 99’ with different cold resistance under 4 °C cold stress, the expression patterns of RRM1 family members in *Brassica rapa* were compared in different periods (Fig. 5, Table S4). The results showed that the expression of RRM1 family members in *Brassica rapa* was significantly different between ‘Longyou 7’ and ‘Longyou 99’ under 4 °C cold stress for 3 h and 24 h. The expression level of Brapa08T000410 in ‘Longyou 7’ was the highest at 3 h and 24 h under 4 °C cold stress, while that of Brapa07T003150 and Brapa09T002178 in the weak cold resistant variety of ‘Longyou 99’, was the highest at 3 h and 24 h under 4 °C cold stress. Under cold stress for 24 h, Brapa05T000840, Brapa06T000422, and Brapa10T002529 were highly expressed in ‘Longyou 7’, while, Brapa05T000840,, Brapa04T002810, and Brapa06T00042 were highly expressed in ‘Longyou 99’.

### Expression analysis of RRM1 genes family in *Brassica rapa* under cold stress and salt stress

**Fig. 6:**
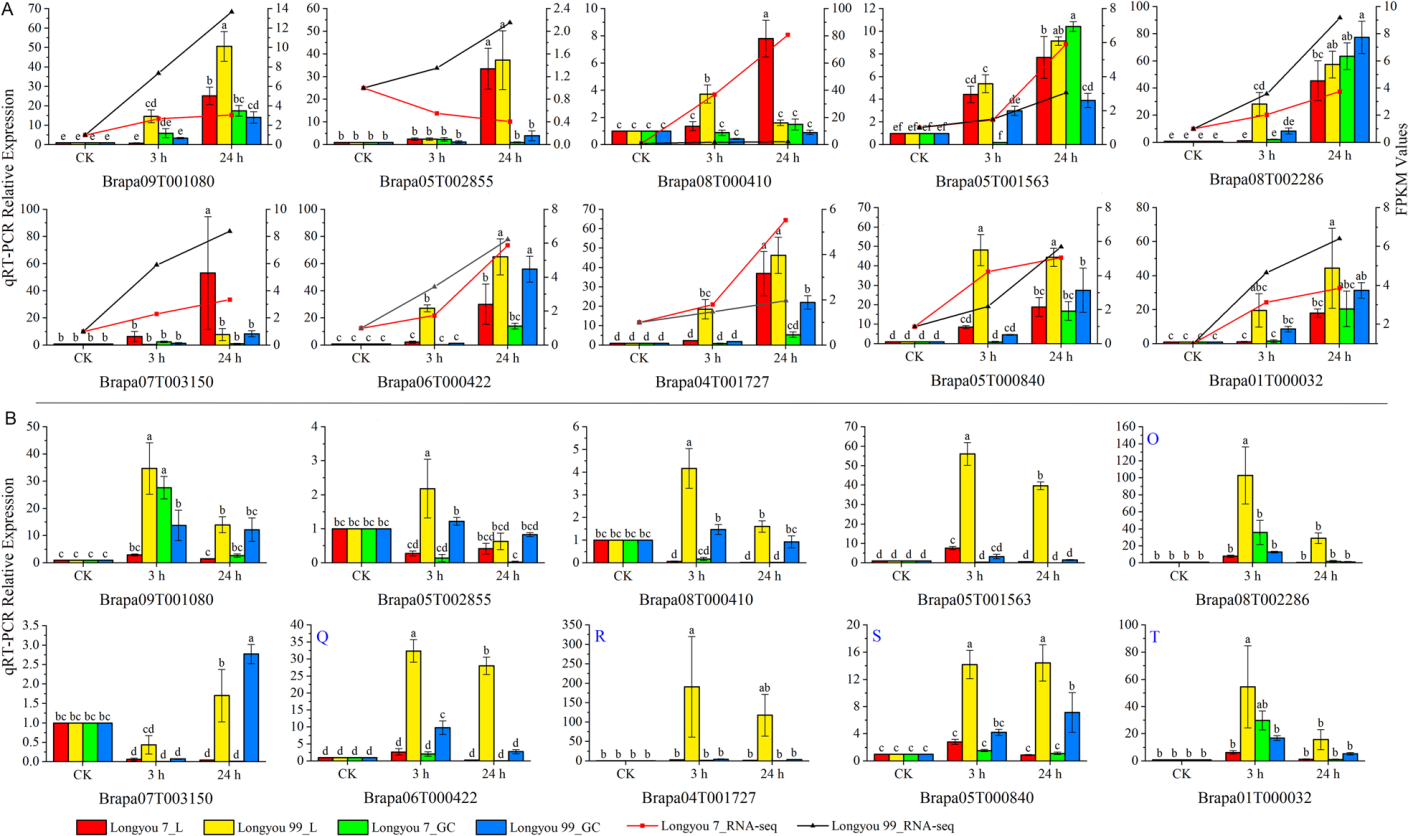
Expression analysis of *BrRBPs* in growth cones (GC) and leaves (L) of strong cold resistance (‘Longyou 7’) and weak cold resistance varieties (‘Longyou 99’) under cold and NaCl stress treatment. A represent cold stress, and B represent NaCl stress. The line graph indicates the FPKM values of the growth cones under cold treatment. The data represents the mean ± standard error of three biological samples, different lowercase letters indicate significant differentiation (*p-value* < 0.05)

Ten differentially expressed genes selected from the transcriptome data were subjected to preliminary detection qPCR. To determine the expression pattern and tissue expression specificity of RRM1 genes in low temperature stress. In addition, to identify and screen salt stress responsive genes, we performed expression pattern analysis of these 10 genes under NaCl stress. The results showed some differences in the expression of the RRM1 gene in different varieties or tissues and times of the same variety at 3 h and 24 h of cold stress 4 °C treatment (Fig. 6A). With the increase of low-temperature treatment time, the expression of the gene showed an increasing trend, and the expression in ‘Longyou 7’ leaves and growth cones were significantly higher than that in ‘Longyou 99’ at 24 h of low-temperature treatment, respectively. The expression of Brapa07T003150, Brapa08T000410, and Brapa05T001563 in ‘Longyou 7’ was significantly higher than that in growth cones at 24 h, while the expression of the remaining seven genes was the opposite. In ‘Longyou 99’, except for Brapa08T000410 and Brapa05T001563, the expression at 24 h was significantly higher in leaves than in growth cones. The expression was significantly higher in growth cones than in leaves. The expression of Brapa05T000840 was higher in both ‘Longyou 7’ and ‘Longyou 99’ leaves and growth cones under 4 °C cold stress treatment for 24 h. Therefore, we selected this gene for further functional validation to elucidate its cold resistance mechanism in winter *Brassica rapa*.

Under NaCl stress for 3 h and 24 h, the gene expression of the RRM1 gene was significantly higher in growth cones than in leaves (Fig. 6B). After 3 h treatment, the expression of the RRM1 gene in ‘Longyou 7’ growth cones was significantly higher than that in ‘Longyou 99’ growth cones and leaves. In ‘Longyou 99’, the expression of Brapa08T002286 was significantly higher in leaves than in growth cones at 3 h, and the expression in growth cones was significantly higher than in leaves.

### Identification of growth and freezing resistance at low temperatures in *BrRBP*-transformed *Arabidopsis thaliana*

**Fig. 7:**
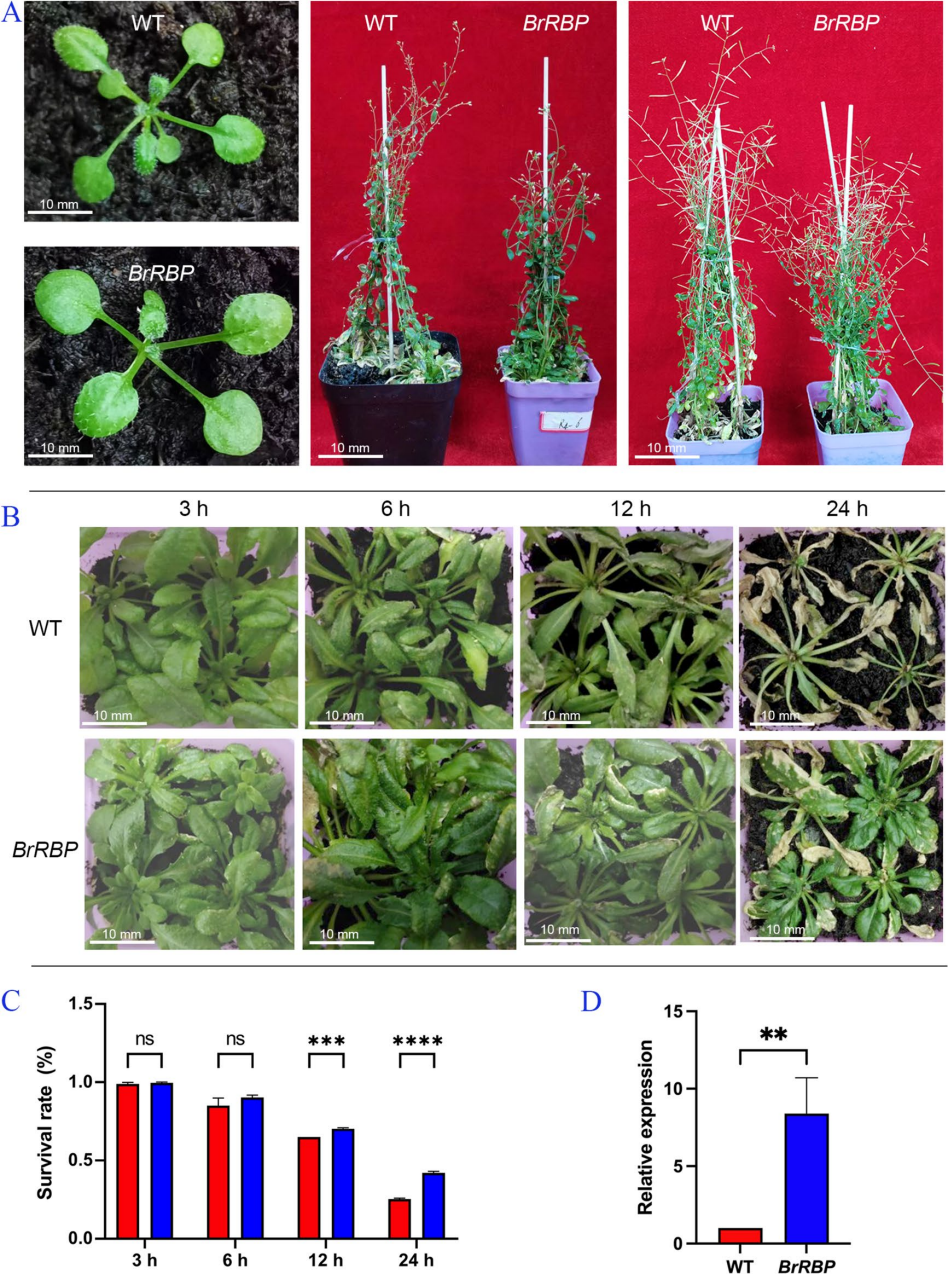
Phenotype and expression of transgenic *BrRBP Arabidopsis* after low-temperature treatment. **A** Phenotypes of transgenic *BrRBP Arabidopsis thaliana* at seedling, flowering, and maturity stages. **B** The phenotype of transgenic *BrRBP Arabidopsis* after low-temperature treatment. **C** The survival rate of *Arabidopsis* plants after low-temperature treatment. **D** qRT-PCR detection of transgenic *BrRBP Arabidopsis*. WT: wild-type *Arabidopsis*, *BrRBP*: *BrRBP* transgenic *Arabidopsis*

DNA was extracted from both transgenic and wild-type *Arabidopsis thaliana*. PCR amplification results showed that wild-type *Arabidopsis* plants had no amplified bands, and transgenic plants amplified bands of about 850 bp. This indicates that the *BrRBP* gene (Brapa05T000840) was successfully transformed (Fig. S1, Table S5). Under the same environmental conditions of temperature and light, the phenotypes of wild-type and overexpressed *Arabidopsis thaliana* were observed. There was no significant difference between the two plants at the seedling stage, and the leaf size was the same. The wild-type plants were earlier at the flowering stage, while the *BrRBP* transgenic plants were stunted and dwarfed. Wild-type *Arabidopsis* set pods early at the maturity stage, while transgenic *Arabidopsis* set pods slowly (Fig. 7A).

To verify the response of the *BrRBP* gene to low-temperature stress, wild-type and transgenic *Arabidopsis* plants were treated with a low temperature of -4 °C for 0 h, 3 h, 6 h, 12 h, and 24 h, respectively, and placed at room temperature for 7 days after removal. The leaves of wild-type and transgenic *Arabidopsis* plants with the *BrRBP* gene showed no significant change after 3 h treatment, and the survival rates were similar. After 6 h of treatment, the leaves began to wither, and the survival rate of wild-type *Arabidopsis* decreased by about 85%, while the survival rate of transgenic plants with the *BrRBP* gene was 90%. At 12 h of low-temperature treatment, the leaves of transgenic and wild-type *Arabidopsis* plants showed signs of yellowing, and the survival rate of transgenic *Arabidopsis* was 70%. Meanwhile, the survival rate of wild-type *Arabidopsis* decreased by about 65%. At 24 h of treatment, the leaves of most wild-type seedlings turned yellow, withered, and died, and the survival rate was only 27%, while leaves of transgenic *Arabidopsis* plants were partially yellowing. Most of them returned to normal growth (Fig. 7B-C). In addition, expression of *BrRBP* transgenic *Arabidopsis* was significantly higher than that of wild-type *Arabidopsis* (Fig. 7D).

### Physiological and biochemical indicators determination of *BrRBP* transgenic plants after low-temperature stress

**Fig. 8:**
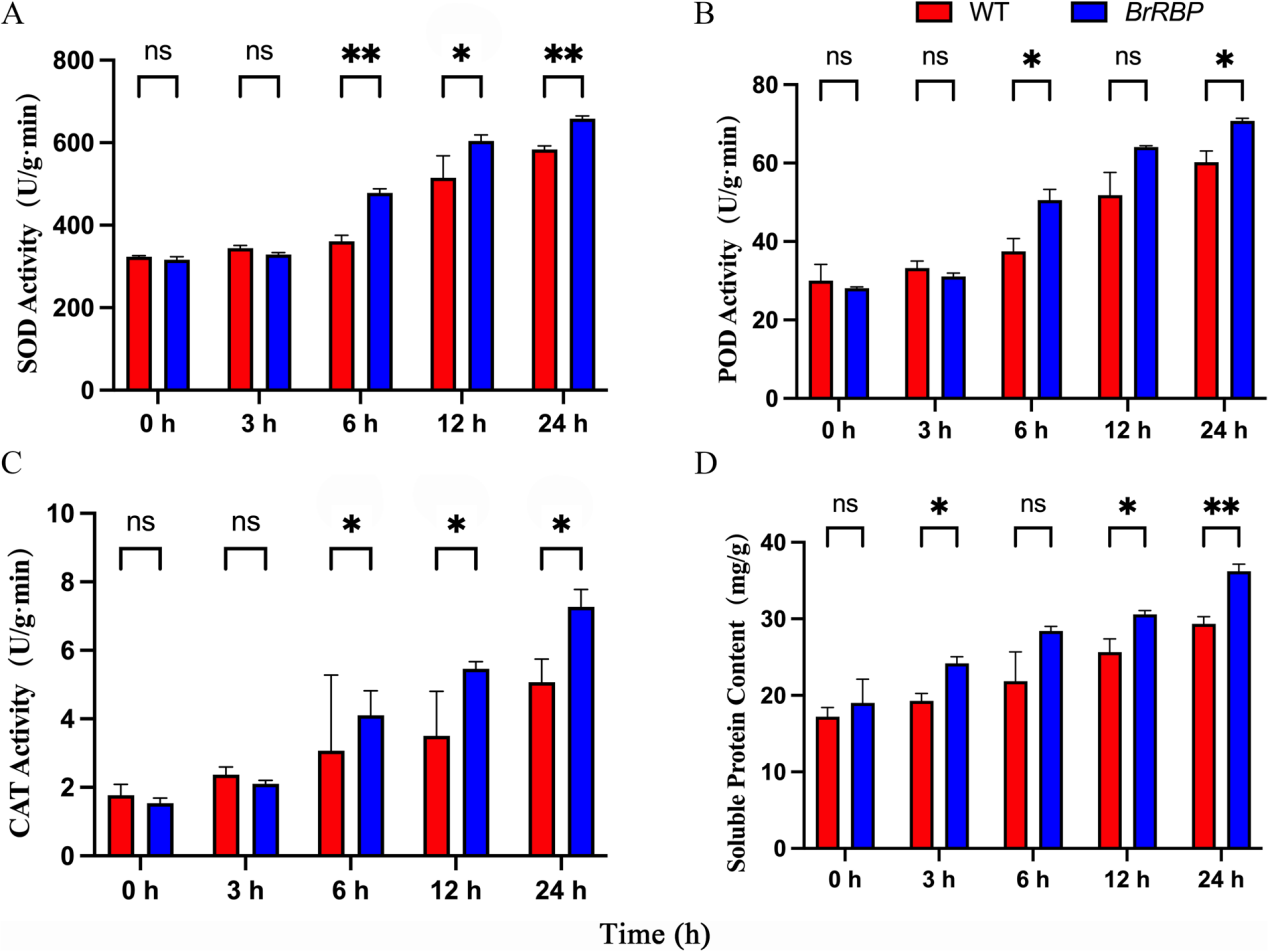
Physiological and biochemical indicators determination of *BrRBP* transgenic plants after low-temperature stress. **A** SOD activity. **B** POD activity. **C** CAT activity. **D** Soluble protein content. WT: wild-type *Arabidopsis*, *BrRBP*: *BrRBP* transgenic *Arabidopsis*

Superoxide dismutase (SOD), Peroxidase (POD) and Catalase (CAT) have important roles in the reactive oxygen species (ROS) scavenging system. When plants are subjected to low-temperature stress, it triggers the accumulation of reactive oxygen species and osmotic substances, leading to changes in antioxidant enzyme activity. To prevent low-temperature induced damage, antioxidant enzymes and non-enzymatic techniques have been developed in plants to eliminate ROS. The transgenic *Arabidopsis thaliana* was treated with low-temperature, we measured the relevant physiological and biochemical indexes, the results showed that, the SOD activity of transgenic *Arabidopsis thaliana* was 3.04 times higher than that of the control and 1.13 times higher than that of wild-type plants treated for 24 h. The SOD activity of wild-type plants reached the maximum at 24 h, 1.80 times higher than that of untreated plants (Fig. 8A). At 24 h, the POD enzyme activity of transgenic *Arabidopsis* was 2.53 times higher than that of untreated *Arabidopsis* and 1.17 times higher than that of wild-type *Arabidopsis*. There was a significant difference in POD activity between transgenic and wild-type *Arabidopsis* at 24 h (Fig. 8B). At 24 h, the enzyme activity of wild-type *Arabidopsis* was 2.01 times higher than that of untreated *Arabidopsis*, the CAT activity of transgenic *Arabidopsis* was 4.74 times higher than that of untreated *Arabidopsis* and 1.43 times higher than that of wild-type *Arabidopsis*, the CAT activity of wild-type *Arabidopsis* was 2.87 times higher than that of untreated *Arabidopsis*, and the difference was significant (Fig. 8C). Also, the soluble protein content of transgenic *Arabidopsis* was 1.62 times higher than that of untreated *Arabidopsis* and 1.23 times higher than that of wild-type *Arabidopsis*, and the soluble protein content of wild-type *Arabidopsis* was 1.77 times higher than that of untreated *Arabidopsis* (Fig. 8D). After low-temperature treatment, the activities of SOD, POD, CAT, and soluble protein in *BrRBP* transgenic *Arabidopsis* plants increased with the extension of treatment time. After 24 h of treatment, all reached the highest value. The activities of four physiological indexes in transgenic plants were higher than in wild-type plants. The results showed that the ROS scavenging ability of *BrRBP* transgenic plants was stronger than that of wild-type plants.

## Discussion

Plants have evolved many specific RNA-binding proteins, and the genome of *Arabidopsis* encodes 196 RRM-containing proteins [33]. This study identified 102 RRM1 gene family members from *Brassica rapa* L, distributed on 10 chromosomes. Phylogenetic relationships showed that the RRM1 family of *Brassica rapa* L. and *Arabidopsis thaliana* was divided into 14 subgroups (I–XIV). The RRM1 family members of *Brassica rapa* and *Arabidopsis thaliana* were unevenly distributed in each subfamily. The genes on the same branch may have similar functions, indicating that the RRM1 gene members of *Brassica rapa* and *Arabidopsis thaliana* were relatively conserved during evolution and had certain functional similarities. Nine conserved protein motifs were predicted from the 102 RRM1 family members. Most members of the same subfamily contain the same Motif composition. Collinearity analysis of the *Brassica rapa* L. genome showed 89 pairs of collinearity genes belonging to segmental duplication. Also, the RRM1 gene family members in *Brassica rapa* have collinearity with the RRM1 gene family members in *Arabidopsis thaliana*, *Brassica napus*, and *Brassica juncea*, also indicating that the RRM1 gene family members in the process of evolution are highly conserved.

The RRM domain is important for the post-transcriptional regulation of gene expression, including RNA processing. In higher plants, RNA-binding proteins have several important functions in pollen development, hormone signaling, and circadian rhythm [34]. Some RRM proteins are involved in plant development and stress response, but the molecular mechanisms of their regulatory roles are rarely studied [6, 35–37]. The *Arabidopsis* protein FCA contains the RRM domain and has been shown to play a role in controlling flowering time [38]. *RBD1*, *CP29A*, and *CPP31A* may promote cold tolerance in *Arabidopsis* through RNA metabolism [6, 39, 40]. The expression of *ORRM5* could accelerate *Arabidopsis* seed germination and seedling growth under cold stress but had no significant response to salt treatment. The expression of the *ORRM5* gene in rice could improve grain yield under drought stress [41]. In this study, we analyzed the expression of the RRM1 gene family under cold stress and salt stress. Under different stress treatments, there were certain differences in the expression of RRM1 gene family members in different *Brassica rapa* cultivars or tissues and times of the same cultivar. With the increase in low-temperature treatment time, the expression of RRM1 gene family members showed an upward trend. At low-temperature treatment for 24 h, the expression levels in ‘Longyou 7’ leaves and growth cones were significantly higher than those in ‘Longyou 99’. At 3 h and 24 h of NaCl stress, the expression of RRM1 gene family members in growth cones was significantly higher than in leaves. At 3 h of treatment, the expression of RRM1 gene family members in ‘Longyou 7’ growth cones was significantly higher than in ‘Longyou 99’ growth cones and leaves. These results indicated that the RRM1 gene family played a certain role in *Brassica rapa* under low-temperature and salt stresses.

In addition, we found Brapa08T000410 showed the highest expression at 3 h and 24 h under 4 °C cold stress in ‘Longyou 7’. Brapa07T003150 and Brapa09T002178 showed the highest expression at 3 h and 24 h under cold stress in ‘Longyou 99’. The expression of Brapa05T000840 was higher in both ‘Longyou 7’ and ‘Longyou 99’ treated with cold stress at 4 °C for 24 h. And transcriptomic analysis under low temperature stress showed that Brapa05T000840 (Bra005212) has more prominent expression at low temperature. And it is involved in pathways such as RNA binding, response to cold, and response to abscisic acid [2, 42]. Interestingly, we found a high expression of Brapa08T002286 and Brapa05T001563 in ‘Longyou 7’ leaves under salt stress, which helped us to further investigate the function of this gene under other abiotic stresses brought insights.

We transformed Arabidopsis with the ‘Longyou 7’ Brapa05T000840 gene to further investigate its cold tolerance function. Wild-type and *BrRBP*-transgenic *Arabidopsis* did not differ significantly in growth at the seedling stage and had the same leaf size. At the reproductive growth stage, wild-type plants flowered and matured earlier than *BrRB*P transgenic plants. This tends to be consistent with the growth characteristics of strong cold-resistant winter rapeseed in the north of China [2, 3, 42]. After low-temperature treatment, the transgenic plant was less damaged by stress relative to the wild type, and its phenotype and growth were less affected after returning to room temperature. *OsGRP1* and *OsGRP4* in rice reversed the growth defect of cold-sensitive *Arabidopsis* grp7 mutant under cold and freezing stress. *OsGRP6* endowed the grp7 mutant with freezing tolerance, and the expression of *AtGRP7* was inhibited, which was sensitive to cold and freezing stress [43].

When plants face low temperature stress, cell damage can delay plant growth or reduce membrane integrity. The acquisition of plant cold tolerance is a complex physiological response process, and various factors can alter cold adaptation ability [44]. In response to low temperature stress, plants initiate a series of signal transduction reactions, reduce tissue water content, accumulate osmotic substances, and change antioxidant enzyme activity levels, thereby improving cold tolerance. Usually, cold stress triggers the accumulation of reactive oxygen species (ROS), such as superoxide free radical O_2_^−^, hydroxyl free radical OH, and hydrogen peroxide H_2_O_2_, to increase oxidative stress in plants. In order to prevent low-temperature induced oxidative damage, all plants have developed methods for clearing ROS through enzymatic antioxidant techniques such as POD, SOD, and CAT, as well as non-enzymatic methods [45–47]. Therefore, they are often used as one of the indicators for screening resistance. In the present work, transgenic BrRBP plants showed a significant increase in the level of their own antioxidant enzyme activity compared to the wild type after being stimulated by stress, which improved the water retention capacity of the cells and avoided cell membrane damage. There are several reports indicating phytohormones and antioxidants can induce the main cell signaling associated with response to abiotic stress such as cold stress and, as a result, ion balance is established and cell damage is reduced [48–50]. It seems that RPM1 is also involved in reducing cold stress injuries and increasing resistance.

In this study, we found that the wilting and damage degree of transgenic *Arabidopsis* leaves was less than that of wild-type *Arabidopsis* after low-temperature treatment, and the survival rate of transgenic *Arabidopsis* was significantly higher than that of wild-type *Arabidopsis*, indicating that *BrRBP* gene indeed improved the cold resistance of plants. After low-temperature treatment, the enzyme activities of transgenic *BrRBP* and wild-type plants were increased, and the increasing trend of enzyme activities of transgenic *BrRBP Arabidopsis* plants was higher than that of wild-type plants. The results showed that the *BrRBP* gene was related to cold tolerance, and transgenic *Arabidopsis thaliana* with the *BrRBP* gene had better cold tolerance.

## Conclusions

In this study, we found that 102 RRM1 gene family members were distributed on 10 chromosomes of *Brassica rapa*, and 9 conserved protein motifs were predicted. Most members of the same subfamily contain the same Motif composition. The results of collinearity analysis in the *Brassica rapa* L. genome showed 89 pairs of collinearity genes belonging to segmental duplication. These genes are highly conserved during evolution. Quantitative analysis showed that the expression of the Brapa05T000840 gene was higher in ‘Longyou 7’ and ‘Longyou 99’ under cold stress at 4 °C for 24 h, indicating that the Brapa05T000840 gene plays an important role in plant growth and development and stress response. Under low temperatures, the survival rate of *BrRBP* transgenic *Arabidopsis* was higher than that of the wild type, and the degree of leaf damage was lower than that of the wild type. The SOD, POD, and CAT activities were higher in transgenic *Arabidopsis* than in wild-type *Arabidopsis*. These results provide a theoretical basis for analyzing the cold resistance mechanism of winter rape (*Brassica rapa*) and improving the cold resistance of northern winter rape (*Brassica rapa*).

## Materials and methods

### Material planting and stress treatments

Two winter *Brassica rapa* varieties, ‘Longyou 7’ (AA, 2n = 20, ultra cold resistance, overwintering rate of 90%) and ‘Longyou 99’ (AA, 2n = 20, weak cold resistance, overwintering rate of 14%), with different freezing resistance, were selected as the experimental materials (provided by the rapeseed breeding group of Gansu Agricultural University, Lanzhou, China). The healthy, full, and uniform seeds were placed in glass Petri dishes with two layers of wet filter paper for germination. After the seeds were exposed, they were transferred to seedling pots filled with substrate and vermiculite (3:1) and cultured in a plant incubator (25 °C, 16 h light/8 h dark cycle). The seedlings were treated with abiotic stress when the rapeseed grew to the six-leaf stage. The seedlings were treated in a 4 °C low-temperature incubator for 3 h and 24 h, respectively, with a normal temperature of 22 °C as the control treatment. The seedlings were treated with NaCl (180 mmol/L) for 3 h and 24 h, respectively, and water was added as the control [26, 27]. Each treatment was set up in three biological replicates. After treatment, the growth cones (dried on filter paper and dissected into slices about 5 mm thick) and leaves of rapeseed were collected quickly, rinsed with distilled water, frozen immediately with liquid nitrogen, and then stored in -80 °C refrigerator for subsequent RNA extraction and reverse transcription tests.

### Identification of the RRM1 gene family and analysis of phylogenetic relationships in *Brassica rapa*

*Brassica rapa* (Version 3.0) and *Brassica juncea* (Version 2.0) genome information were downloaded from the BRAD Database (http://brassicadb.cn/) website, *Brassica napus* (ZS11.v10) genome information were downloaded from the BnIR Database (https://yanglab.hzau.edu.cn/BnIR/genome_data), and *Arabidopsis thaliana* genome information from the *Arabidopsis* Database TAIR (https://www.arabidopsis.org/) website. Our group has obtained a high quality genome for the winter *Brassica rapa* ‘Longyou 7’, and used it as a reference genome for analysis [42]. We downloaded the hidden Markov model file for the RRM_1 domain (PF00076) from the Pfam database (http://pfam.sanger.ac.uk/) website [51]. The HMM(Hidden Markov Mode) model was constructed using the HMMER 3.1 software (http://hmmer.org/download.html) website, and the RRM1 members were searched in the *Brassica rapa*, ‘Longyou 7’, *Brassica juncea*,* Brassica napus*, and *Arabidopsis* proteome database (E-value ≤ 1e-5) [52]. The candidate members were submitted to the online websites SMART (http://smart.embl-heidelberg.de/), pfam (https://www.ebi.ac.uk/interpro/) and CD-Search (https://www.ncbi.nlm.nih.gov/Structure/cdd/wrpsb.cgi) for manual screening, and the redundant sequences were deleted [53–57], and 102 RRM1 members of *Brassica rapa* and 71 RRM1 members of *Arabidopsis thaliana* were obtained. The RRM1 protein sequences of *Brassica rapa* and *Arabidopsis thaliana* were aligned using ClustalW, and the phylogenetic evolutionary tree was constructed via the maximum likelihood method with 1000 bootstrap replicates using MEGA 7.0 software [58–60].

### Sequence structure, protein physicochemical properties, chromosomal localization, and collinearity analysis of RRM1 gene family members

The conserved motif types in RRM1s protein sequences were analyzed using MEME (http://alternate.meme-suite.org/tools/meme) software [61]. The prediction and analysis of the protein amino acid length, theoretical isoelectric point, relative molecular mass, and other indicators of RRM1 gene family members was completed using ProtParam of the online software ExPASy (https://web.expasy.org/protparam/) [62]. Subcellular localization analysis was conducted using the Plant module of the online software WOLF PSORT (https://wolfpsort.hgc.jp/) [63]. The structures of exons and introns of RRM1 gene family members were analyzed via Gene Structure Display Server software (GSDS2.0, http://gsds.cbi.pku.edu.cn) [64]. Based on the location information of the RRM1 gene on chromosomes, the chromosome location map of the RRM1 gene was drawn using MapChart software [65]. The repetitive events and collinear relationship among RRM1 genes in *Brassica rapa* were analyzed using MCScanX and plotted using Tbtools software [66, 67]. There are two types of genes in a collinearity relationship: tandem duplication and segmental duplication. When the alignment rate and similarity of the two genes were greater than 70%, and their position spacing on the chromosome was less than 100 kb, the two genes were considered to be originated from tandem duplication [68, 69].

### Analysis of the expression pattern of RRM1 gene family members

The total RNA of rape ‘Longyou 7’ and ‘Longyou 99’ growth cones and leaves were extracted using Trizol Regent (DP419, Tiangen Biology Co., Ltd., Beijing, China) kit according to the manufacturer’s instructions, and its purity was tested via 1% agarose gel electrophoresis. The first strand of cDNA was synthesized via reverse transcription according to the instructions of the PrimeScript® RT kit (RR036A, TaKaRa, Dalian, China). The quality and concentration were detected via an ultra-trace ultraviolet–visible spectrophotometer (Danol, Spanish Fort, AL, USA). The samples were stored in a refrigerator at -80 °C.

In total, 10 candidate genes with different expressions in the transcriptional group were selected, and specific primers were designed (Table S6). Real-time fluorescence quantitative PCR was performed under low-temperature and salt stresses. We used SYBR Premix Ex Taq TM II (RR820A, TaKaRa, Beijing, China) for real-time fluorescence quantitative PCR (qRT-PCR) analysis on LightCycler 96 (Roche, Mannheim, Germany). The reaction system and procedure were performed according to the manufacturer’s instructions. The actin gene of *Brassica rapa* was used as the internal control to standardize the expression of the cDNA template. The relative expression of each gene was calculated via the comparison 2^−ΔΔCT^ method. The RNA-seq library (SRP179662) under cold stress was selected for gene expression analysis, control plants were then grown in a growth chamber under normal conditions (22 ◦C with 16/8 h light/dark cycle), For cold treatments, plants were transferred to a 4 ◦C growth chamber (Yuejin, Shanghai, China). The growth points of plants subjected to 22 ◦C (as control) and 4 ◦C for 3 h, 24 h (as treatments) were collected and frozen in liquid nitrogen and stored at −80 ℃, then used for RNA-seq. Each sample were pooled from three plants and three biological replicates were included [2, 3].

### Genetic transformation of *BrRBP* gene in *Arabidopsis thaliana*

The *BrRBP* (RNA binding protein CP29b, Brapa05T000840) gene of *Brassica rapa* was cloned by PCR based on specific primers (*BrRBP*-F: CTATCCTTCGCAAGACCCTTC, *BrRBP*-R: AAACTGGCGCCTTGGAGGC). With reference to the Gateway technology, PCR amplification product was obtained using primers (*BrRBP*-J-F: AAAAAAGCAGGCTTCCTATCCTTCGCAAGACCCTTC, *BrRBP*-J-R: AGAAAGCTGGGTC AAACTGGCGCCTTGGAGGC), The recovered product was connected with BP Clonase enzyme (Invitrogen, Carlsbad, CA, USA) and pDONR vector. After transformed into *Escherichia coli* (DH5α). the positive clones were selected to connect with LR Clonase enzyme (Invitrogen, USA) to the overexpression vector 35S: pEarly-Gate101-YFP. *Agrobacterium tumefaciens* transformed GV3101(Anyu Biotechnology Co., Ltd., Shanghai, China); After the bacteria were picked out from the coated plate, colony PCR was carried out and the positive bacterial solution was selected and preserved.

We activated the *Agrobacterium bacterium* solution, measured OD at 600 nm, adjusted the OD of the solution to 0.8, and added a surfactant. The inflorescence of *Arabidopsis thaliana* was dipped in the bacterial solution for 1 min, and the excess bacterial solution was wiped off from other parts of the plant. The plant was kept for 24 h away from light, photo-cultured three times at 1-week intervals, and harvested after maturation [70, 71].

We sprayed the plant with Herbicide glufosinate ammonium (Basta, 1/10000) (Sangon Biotecch, shanghai, China) for screening [72]. T1 generation seeds were sown in culture substrate, sprayed with herbicide three times, screened for surviving plants and transplanted, and harvested singly to obtain T2 generation seeds. T2 generation seeds were spot sampled in 1/2 MS medium containing extracted and sterilized herbicide Basta (1/10000), and dark treated at 4 °C for 3 days. After 10 days, T3 generation seeds were screened according to Mendel’s 3:1 law of segregation, and seeds were obtained singly. T3 generation seeds continued to be spotted in 1/2 MS medium containing the herbicide (filter sterilised) Basta. Plants that all survived and did not turn yellow were considered homozygous generation transgenic lines.

The pure transgenic *Arabidopsis* plants were placed in a 4 ℃ incubator and treated for 3 h, 6 h, 12 h, and 24 h. Some of the plants were sampled for RNA extraction and physiological index determination, and the others were returned to the growth chamber at 23 °C. The number of surviving plants was observed and counted after 7 days to calculate the survival rate. Primers *RBP-F*: CTCCGTCTTCTCTTCCTCCTCCTC; *RBP-R* AACTGCCGTCTTCTTCTTCTTCGACAG were used for qRT-PCR assay on *Arabidopsis*; primer *Actin-F*: ACAGCGAGAGAAAGTAGCAGA. *Actin-R*: TTGATAAGAGCGGTCCATTTGAA was used as an internal reference.

The activity of superoxide dismutase (SOD) was determined using the reference tetrazolium file blue (NBT) photoreduction method [73, 74]. The activity of peroxidase (POD) was determined using the guaiacol method [75]. The activity of catalase (CAT) was determined via the UV absorption method, and the soluble protein content was determined via the Komas Brilliant Blue method [76].

### Data processing and analysis

Excel 2016 and SPSS 21 (SPSS Inc., Chicago, IL, United States of America) were used for data statistics and analysis. One-way ANOVA and Duncan’s method were used for significant difference analysis; the significance level was *P* < *0.05* [50].

### Supplementary Information

**Additional file 1: Table S1.** Basic physicochemical properties of RRM1 amino acids.

**Additional file 2: Table S2.** Motif prediction of RRM1 protein by MEME server.

**Additional file 3: Table S3.** Collinearity of the RRM1 gene in *Brassica rapa*, *Arabidopsis thaliana*, *Brassica napus*, and *Brassica juncea*.

**Additional file 4: Table S4.** Log2 means of FPKM of different cold-resistant varieties in RNA-seq under cold stress for RRM1.

**Additional file 5: Table S5. ***Brassica rapa* Brapa05T000840 sequence.

**Additional file 6: Table S6.** Physicochemical Properties of BrRRM1s Amino Acids.

**Additional file 7: Fig. S1.** Identification of *BrRBP* transgenic *Arabidopsis* positive plants.

## Data Availability

Raw sequencing reads generated in this study were obtained from the National Center for Biotechnology Information (NCBI) with SRA ID: SRP179662 (https://www.ncbi.nlm.nih.gov/sra/?term=SRP179662), doi:10.3390/ijms20051071.
