# Supplementary material for: Genome-wide identification of RNA recognition motif (RRM1) in Brassica rapa and functional analysis of RNA-binding protein (BrRBP) under low-temperature stress
Source: BMC Plant Biol. 2023 Dec 7;23:621. doi: 10.1186/s12870-023-04639-4 (PMC10701981; doi:10.1186/s12870-023-04639-4)
Supplement: Supplementary file 5 — Additional file 5: Table S5. Brassica rapa Brapa05T000840 sequence. [file 12870_2023_4639_MOESM5_ESM.docx]

**Supplementary Table S5. Brassica rapa Brapa05T000840 sequence**

> Brapa05T000840 cds:protein_coding

ATGGCTGCTTCAGCTTCCTCTCTCGCTCTCTCCACCTTCAACCCCAACTCCCTTCCTTTC

TCCGTCTCCAGACCCTCCTCCCTCTTACCTCCTTCCGTCTCCTTCAAACTCAACTCCGTC

TTCTCTTCCTCCTCCTCCGCCAAATGCACCTCTCCCGCCTCTCGCTTCCTCCGTAACGTC

GCCGTCACGGAAGACTTCTCTGTCGAAGAAGAAGACGGCAGTTTCGCCGACGACGCACCG

CCGCCGCCGCAGGAGCAGTCCTTCTCCGCTGACCTCAAACTCTTCGTCGGTAACCTTCCG

TTTAACGTCGACAGTGCTCAGCTCGCTCAGCTGTTCGAGAGCGCAGGGAACGTTGAGATG

GTTGAGGTAATCTATGACAAGGTGACAGGAAGAAGCAGAGGTTTTGGGTTCGTGACTATG

TCTTCTGTCTCTGAAGTTGAGGCAGCTGCTCAGCAGTTCAATGGCTATGAGTTGGATGGT

AGACAATTGAGAGTTAACGCGGGTCCTCCACCACCAAAGAGGGAAGACTCCTTCTCCAGA

GGACCAAGAAGCAGCTTTGGAGGTGGCGGCGGTGGTGGTGGTGGCTCAGGAAACCGTGTT

TACGTTGGTAATCTCTCGTGGGGAGTCGATGACATGGCTCTGGAGAGTTTGTTTGGGGAG

CAAGGAAAGGTTGTTGAGGCTAGAGTTATCTACGACAGGGACAGTGGTCGGTCCAAGGGT

TTTGGATTTGTCACTTACAACTCTGCTCAAGAGGTTCAAAACGCTATCCAAACCTTGAAT

GGTGCTGATTTGGACGGGAGACAAATTAGAGTGTCGGAAGCTGAGGCTAGGCCTCCAAGG

CGCCAGTTTTGA
