# Supplementary material for: Genome-wide identification of RNA recognition motif (RRM1) in Brassica rapa and functional analysis of RNA-binding protein (BrRBP) under low-temperature stress
Source: BMC Plant Biol. 2023 Dec 7;23:621. doi: 10.1186/s12870-023-04639-4 (PMC10701981; doi:10.1186/s12870-023-04639-4)
Supplement: Supplementary file 6 — Additional file 6: Table S6. Physicochemical Properties of BrRRM1s Amino Acids. [file 12870_2023_4639_MOESM6_ESM.docx]

**Table S6 Primer sequence information of RRM1 gene family**

| Gene ID | Forward primer sequence 5’-3’ | Reverse primer sequence 5’-3’ |
| --- | --- | --- |
| Bra014162 | AGCCTTACTTGGTCGTGACATTCG | CCACCACCGCTTCTGAAGTTACC |
| Bra037056 | TGATGAGACCGCTGCTAATACTGC | ACCACCACCACCTCCTCCATAC |
| Bra032269 | CACTGGAACTTCACGGCACATCC | ACCTCCACCACCACCGCTTC |
| Bra030298 | ATGCGTCTCTGGCTTGCCTTATTC | AATGGCACGGTTAGCATCTTCTGG |
| Bra005212 | CTCCGTCTTCTCTTCCTCCTCCTC | AACTGCCGTCTTCTTCTTCGACAG |
| Bra011869 | CATCACCGTCAACGAGGCTCAG | ATCCTCCACCTCCACCGTAACC |
| Bra010693 | TACCGTCAACGAGGCTCAGTCTAG | AACCACCTCCTCCACCGTAACC |
| Bra003915 | GGCGTGGTGCTCCTAACATGC | GCTGCTTCCACTCCGTTCATCC |
| Bra030284 | GCATCACTGTGAACGAGGCTCAG | AACCACCACCACCTCCGTATCC |
| Bra027167 | ACGTCAACTTCGATTCCGCCATC | CACTACGACGACGGTACGCAAC |
| β-actin | TGTGCCAATCTACGAGGGTTT | TTTCCCGCTCGGCTGTTGT |
