# Supplementary material for: Genome-wide identification of RNA recognition motif (RRM1) in Brassica rapa and functional analysis of RNA-binding protein (BrRBP) under low-temperature stress
Source: BMC Plant Biol. 2023 Dec 7;23:621. doi: 10.1186/s12870-023-04639-4 (PMC10701981; doi:10.1186/s12870-023-04639-4)
Supplement: Supplementary file 7 — Additional file 7: Fig. S1. Identification of BrRBP transgenic Arabidopsis positive plants. [file 12870_2023_4639_MOESM7_ESM.docx]

**
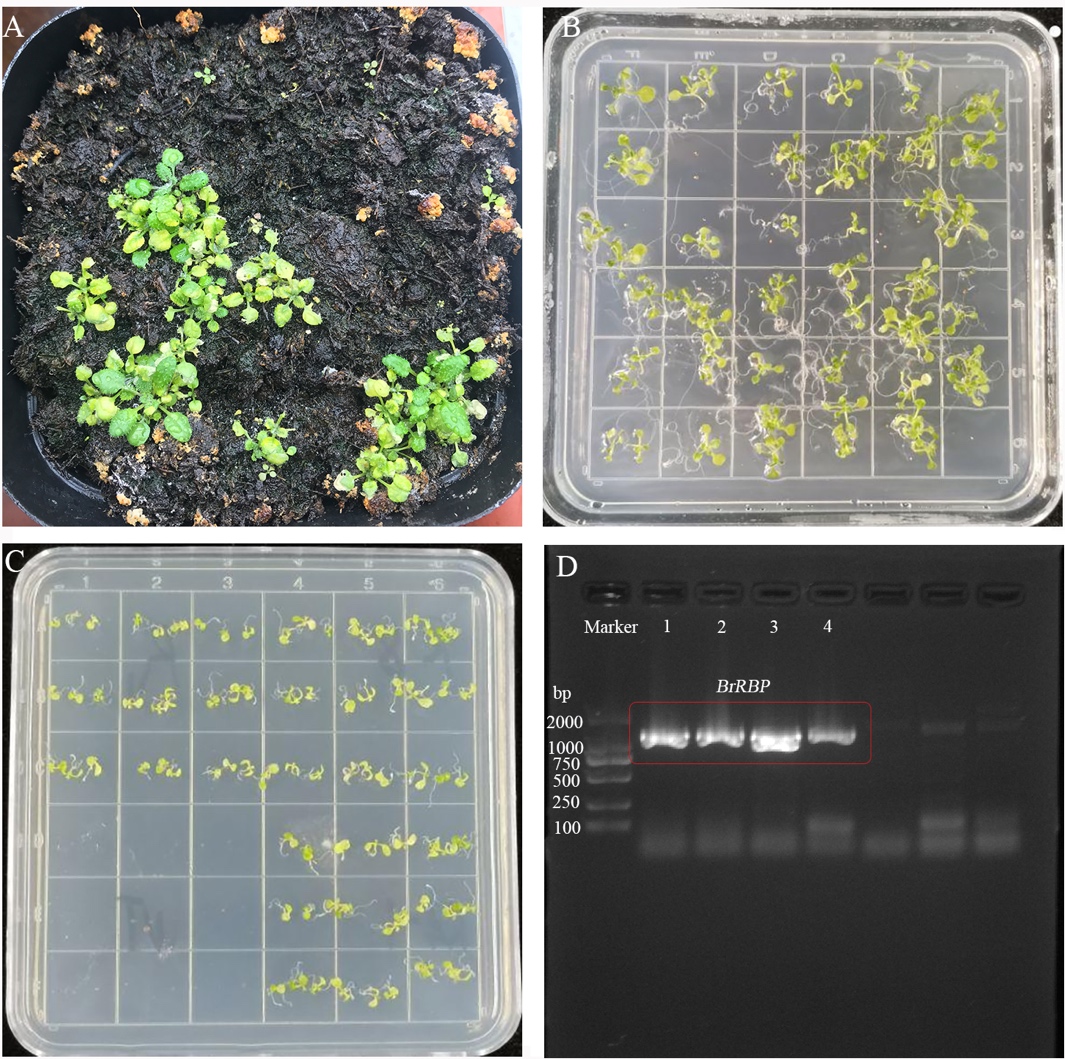
**

Fig. S1 Identification of BrRBP transgenic Arabidopsis positive plants. (A) Growth of the T1 generation of transgenic Arabidopsis thaliana. (B) Growth of the T2 generation of transgenic Arabidopsis thaliana. (C) Growth of the T3 generation of transgenic Arabidopsis thaliana. (D) Gel electrophoresis identification of positive clones in *Arabidopsis* wild-type plants and *BrRBP*-overexpressing transgenic plants.


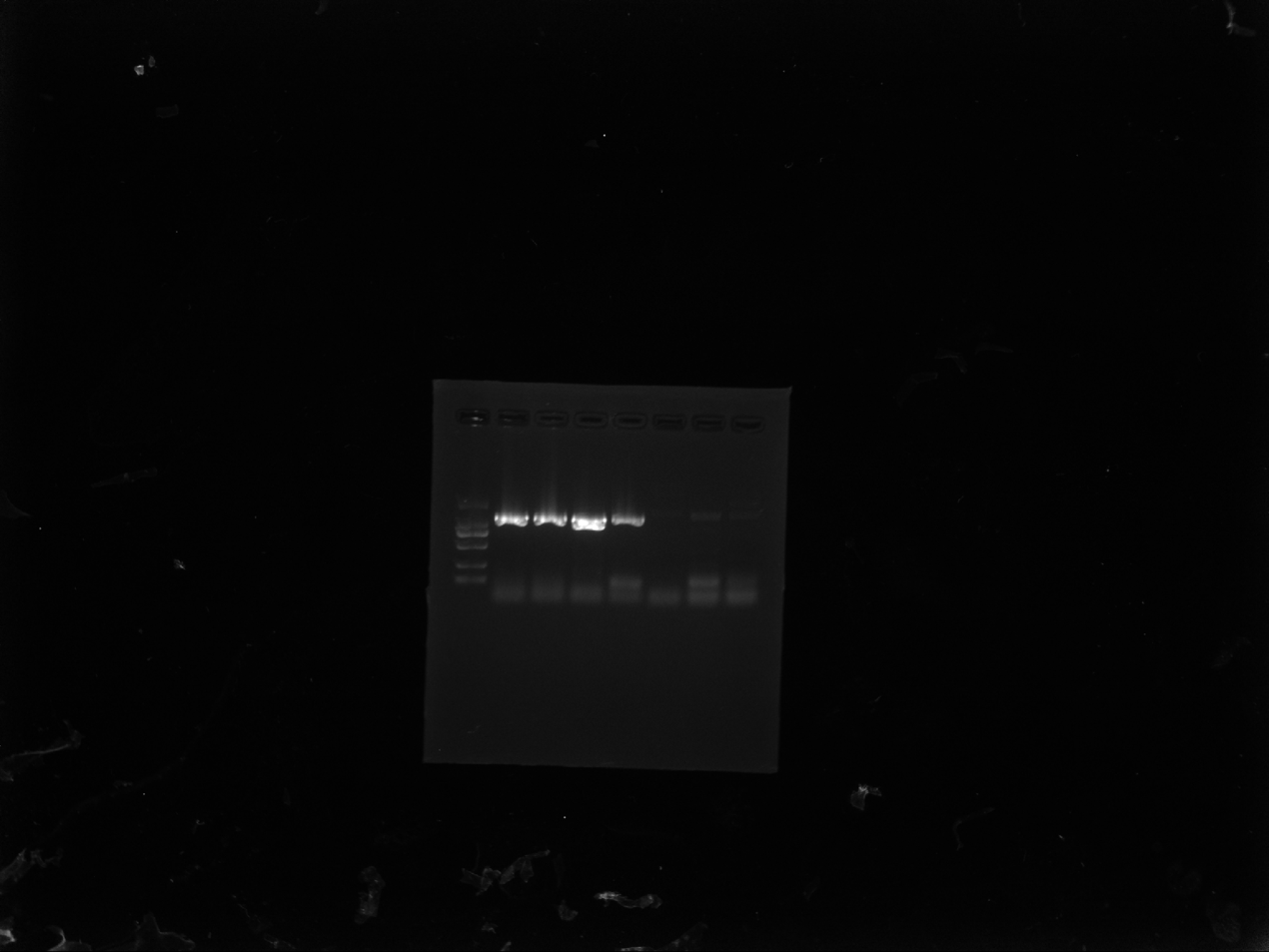


Fig. S1D Original gel electrophoretic identification of positive clones in *Arabidopsis* wild-type plants and transgenic plants overexpressing *BrRBP*.
